# 

**Published:** 1941

**Authors:** 


					r0l. LVIII. No. 218.
INDEXED
SPRING, 1941.
The Bristol
Medico-Chirurgical Journal
PUBUSHBD UNDER THE AU8PI0ES OF
THE BRISTOL MEDICO-CHIRURGICAL SOCIETY.
Editor: J. A. NIXON, C.M.G., M.D., F.R.C.P.
WITH WHOM ABB ASSOCIATED
E. W. HEY GROVES, D.So., M.S., F.R.C.S.
A. E. ILES, O.B.E., M.B., B<S>, F.R.C.S.
A. RENDLE SHORT, M.D., B.S., B.Sc., F.R.C.S.
W. MELVILLE CAPPER, M.B., B.S., F^.S. a ; v
T. M. LING, M.D., MJR.0.F# tyi crQ ? CA
E. WATSON-WILLIAMS, M.C., Ch.M? F.R.O&., Assistant Editor.
ti r. ? ir" ?) 1 *v
> ? ii iiHlJi Ci O '
11/
BRISTOL:
J' W. ARROWSMITH LTD., QUAY STREET & SMALL STREET
Price Three Shillings net.
'
3P ? .s ... ^ SB r*v: ,/
Ssuezssazs: i&a&am

				

